# Unusual aerobic performance at high temperatures in juvenile Chinook salmon, *Oncorhynchus tshawytscha*

**DOI:** 10.1093/conphys/cow067

**Published:** 2017-01-04

**Authors:** Jamilynn B. Poletto, Dennis E. Cocherell, Sarah E. Baird, Trinh X. Nguyen, Valentina Cabrera-Stagno, Anthony P. Farrell, Nann A. Fangue

**Affiliations:** 1 Department of Wildlife, Fish and Conservation Biology, University of California, Davis, CA 95616, USA; 2 United States Environmental Protection Agency, Region 9, 75 Hawthorne Street, San Francisco, CA 94105, USA; 3 Department of Zoology and Faculty of Land and Food Systems, University of British Columbia, Vancouver, BC, CanadaV6T 1Z4

**Keywords:** Conservation, fish, metabolic rate, metabolism, physiology, plasticity

## Abstract

Understanding how the current warming trends affect fish populations is crucial for effective conservation and management. To help define suitable thermal habitat for juvenile Chinook salmon, the thermal performance of juvenile Chinook salmon acclimated to either 15 or 19°C was tested across a range of environmentally relevant acute temperature changes (from 12 to 26°C). Swim tunnel respirometers were used to measure routine oxygen uptake as a measure of routine metabolic rate (RMR) and oxygen uptake when swimming maximally as a measure of maximal metabolic rate (MMR) at each test temperature. We estimated absolute aerobic scope (AAS = MMR − RMR), the capacity to supply oxygen beyond routine needs, as well as factorial aerobic scope (FAS = MMR/RMR). All fish swam at a test temperature of 23°C regardless of acclimation temperature, but some mortality occurred at 25°C during MMR measurements. Overall, RMR and MMR increased with acute warming, but aerobic capacity was unaffected by test temperatures up to 23°C in both acclimation groups. The mean AAS for fish acclimated and tested at 15°C (7.06 ± 1.76 mg O_2_ kg^−1^ h^−1^) was similar to that measured for fish acclimated and tested at 19°C (8.80 ± 1.42 mg O_2_ kg^−1^ h^−1^). Over the entire acute test temperature range, while MMR and AAS were similar for the two acclimation groups, RMR was significantly lower and FAS consequently higher at the lower test temperatures for the fish acclimated at 19°C. Thus, this stock of juvenile Chinook salmon shows an impressive aerobic capacity when acutely warmed to temperatures close to their upper thermal tolerance limit, regardless of the acclimation temperature. These results are compared with those for other salmonids, and the implications of our findings for informing management actions are discussed.

## Abstract (precis)

Aerobic metabolic capacity was similar between juvenile Chinook salmon reared at 15 and 19°C and showed little change with acute warming to 23°C.

## Introduction

Temperature is well known to affect the behaviour and physiology of fishes both directly and indirectly, influencing the geographical distribution of a species as well as specific physiological processes, such as metabolic rate and growth (Fry, 1971; Schmidt-Nielsen, 1999; Moyle and Cech, 2002). As such, understanding how changes in environmental temperature influence early life-history stages in fishes is fundamental in predicting subsequent size- and condition-dependent processes, such as survival, dispersal and migration (Sogard, 1997; Hurst and Conover, 1998; Edeline *et al*., 2006; Brodersen *et al*., 2008). Ultimately, the daily activities of fishes require the metabolic consumption of oxygen, which is a temperature-mediated process (Clarke and Johnston, 1999; Gillooly *et al*., 2001). Therefore, it is important to assess directly the need and capacity to deliver oxygen to active tissues in fishes; information that can then be used as ecologically relevant measures of fish performance.

Metabolic rate of fishes is dependent on temperature and can be assessed directly by measuring standard metabolic rate (SMR). Furthermore, as fish approach their maximal swimming capacity, they tend to increase their aerobic metabolic rate to a maximal metabolic rate (MMR). The aerobic capacity to deliver oxygen to tissues above a basic need can then be calculated from these measures [calculated either by subtracting SMR from MMR, termed absolute aerobic scope (AAS = MMR − SMR), or by dividing MMR by SMR, termed factorial aerobic scope (FAS = MMR/SMR)]. Absolute aerobic scope defines the maximal aerobic capacity available at a given temperature to perform the activities essential for survival that extend beyond routine maintenance of life to include ecologically relevant and important functions (i.e. swimming, foraging, growth, etc.; Pörtner and Knust, 2007; Pörtner and Farrell, 2008; Clark *et al*., 2013). However, it does not predict when or how these activities are used (Farrell, 2016). The oxygen- and capacity-limited thermal tolerance (OCLTT) hypothesis (Pörtner, 2001; Pörtner and Knust, 2007; Pörtner and Farrell, 2008) addresses this by stating that the extremes of the thermal tolerance of an animal will be determined by the aerobic metabolism of active tissues (once an animal is no longer able to supply oxygen to active tissues above a maintenance level, the animal will no longer be able to tolerate temperatures above or below this limit). The OCLTT hypothesis has emerged as a conceptual model to assess thermal performance of aquatic animals and to determine the fundamental thermal range for a particular species (Pörtner, 2001; Pörtner and Knust, 2007; Pörtner and Farrell, 2008), but not without some debate (e.g. Clark *et al*., 2013; Farrell, 2013; Pörtner and Giomi, 2013; Norin *et al*., 2014).

As with many measures of physiological performance, metabolic rates and aerobic scope values are not static and can be modified by biological variables, such as ontogeny (Oikawa *et al*., 1991; Clarke and Johnston, 1999), or environmental variables, such as time of year (Beamish, 1964; Karås, 1990) or thermal history (e.g. Johnston and Dunn, 1987). Consequently, thermal tolerance limits and the optimal temperature range for peak thermal performance can shift with thermal acclimation, e.g. the optimal temperature range is shifted to warmer temperatures with warm acclimation (see Fry, 1971; Stillman, 2003; Schulte *et al*., 2011; Huey *et al*., 2012; Pörtner, 2012; Claireaux and Chabot, 2016). Physiological plasticity is important for the persistence of individuals, hence the population, because it allows for continued physiological performance in the face of changing environmental conditions regardless of whether these changes may be predictable, such as temperature changes that accompany seasonal shifts (Johnston and Dunn, 1987; Pörtner, 2012), or unpredictable, such as those observed as a result of climate change (Stillman, 2003; Seebacher *et al*., 2015). Likewise, populations may have the ability to adapt to new environmental conditions over generations, which can result in local adaptation in the thermal physiology of a species (Fangue *et al*., 2006; Angilletta, 2009; Farrell and Franklin, 2016). Variation in aerobic scope among individuals of a population can mediate population persistence, as some individuals outcompete others, leading to variation in measures of fitness (Farrell *et al*., 2008; Auer *et al*., 2015). Thus, it is important to characterize aerobic scope both for individuals with different acclimation histories and among populations, because the degree of plasticity exhibited by populations or individuals can vary.

As fish populations decline (e.g. Moyle and Leidy, 1992; Moyle *et al*., 2011; Quiñones and Moyle, 2014) and management becomes more crucial for population persistence, knowledge of thermal limits, optimal thermal ranges, and a mechanistic understanding of how key physiological processes, such as metabolic rate, change in response to environmental variables become more important (Beitinger *et al*., 2000; Pörtner, 2001; Niklitschek and Secor, 2005; Horodysky *et al*., 2015; Martin *et al*., 2015; Jeffries *et al*., 2016; Komoroske *et al*., 2016). Therefore, our objective was to estimate the aerobic scope of juvenile Chinook salmon (*Oncorhynchus tshawytscha*) acclimated to two different rearing temperatures (15 or 19°C) and tested over a range of acute temperature changes (12–26°C). We hypothesized that aerobic scope would be maximized over an ecologically relevant thermal range, and that this thermal range would differ between acclimation groups. In the Central Valley of California, Chinook salmon populations are the focus of many important conservation and management programmes (National Marine Fisheries Service, 2009), and state water management programmes are geared towards optimizing conditions for salmonid spawning and migration. In addition, both the acclimation temperatures and the range of test temperatures evaluated can be experienced by juvenile salmon during early development (Sacramento River Temperature Task Group, 2014) and throughout their migration (Interagency Ecological Program Environmental Monitoring Program, 2015). Therefore, it is important to understand how Chinook salmon respond to environmental variables, such as temperature, and how physiological performance can be affected by thermal history. Understanding the variation associated with thermal performance both within and among populations can help further our understanding of how this species is affected by critically important environmental variables, such as temperature.

## Materials and methods

### Fish transport and acclimation

All fish care and protocols were reviewed and approved by the University of California Davis (UC Davis) Institutional Animal Care and Use Committee (protocol no. 18196). Juvenile autumn-run Chinook salmon (*n* = 300) were transported from the California Department of Fish and Wildlife's Mokelumne River Hatchery (Clements, CA, USA) to the UC Davis Center for Aquatic Biology and Aquaculture in early May 2015. Fish were transported in fresh well water in an aerated transport tank that maintained oxygen levels >80% of air saturation. Prior to rearing in acclimation temperatures, fish were held at UC Davis in outdoor flow-through (3 l min^−1^), tanks 1.5 m in diameter supplied with water (13°C) from a fresh, non-chlorinated well, and fish were fed daily to satiation with pelleted trout diet (mix of 2 mm Skretting commercial trout feed and 1.2 mm Rangen sturgeon feed).

Thirty individual fish were randomly transferred to one of nine replicate indoor flow-through tanks 1.5 m in diameter (*n* = 4 and *n* = 5 replicate tanks for 15 and 19°C acclimation groups, respectively). After transfer, fish were given a 7 day initial recovery period, and water temperatures were then increased at a rate of 1°C day^−1^ to either 15 or 19°C. They were held at these acclimation temperatures for a minimum of 3 weeks prior to experiments. Water temperatures were controlled by mixing ambient (18°C) and chilled water (9°C) for the 15°C tanks, and by using 800 W titanium heaters (model TH-0800; Finnex, USA) and temperature controllers (model 72; YSI, OH, USA) for the 19°C tanks. Mean water temperatures (±SEM) for each acclimation group were 14.8 ± 0.06 (*n* = 4 replicate tanks) and 19.3 ± 0.09°C (*n* = 5 replicate tanks). Once acclimation temperatures were reached, each tank of fish was fed at a rate of 2.0% body mass per fish day^−1^, and absolute feed amounts were adjusted every 10–14 days to account for fish growth. There were no differences in body mass between the two acclimation groups; fish mass was 19.8 ± 0.02 g, and fish total length was 12.0 ± 0.01 cm.

### Oxygen consumption measurements

#### Experimental design

Measurements of oxygen uptake were taken over a range of acute temperature changes in 1°C increments from 12 to 26°C. Fish tested at each temperature increment were randomly selected from replicate tanks. Each individual was tested for RMR and MMR once at one temperature, and four fish from each acclimation group were tested at each test temperature. Each test temperature was evaluated using at least one fish from each replicate acclimation temperature tank.

#### Swim tunnel respirometry

Fish were tested in one of three 5 litre automated swim tunnel respirometers (Loligo, Denmark), two of which were controlled using a single computer system (two-tunnel system) and one of which was controlled using a separate system (single-tunnel system). Therefore, during any run, two tunnels were set at the same test temperature and the other tunnel at a different temperature. Acclimation and test temperatures were randomized between the two systems. Water in each swim tunnel was pumped (model 18B; Danner) from a designated sump unique to each system into an aerated water bath surrounding the swim tunnel and was returned to the sump after circulation through the system. Sump water was continuously refreshed with fresh water from a designated non-chlorinated well and was supplied with air stones for additional aeration. The temperature in the respirometers was controlled by circulating water through a chiller (model DSHP-7; Aqua Logic Delta Star) and pumping it back to the sump using a high-volume water pump (model SHE1.7; Sweetwater, USA). In addition, each sump contained two 800 W titanium heaters (model TH-0800; Finnex, USA) connected to variable temperature controllers (model 72; YSI). These two methods were used simultaneously to achieve water temperature control within the swim tunnels with a precision of ±0.5°C. Swim tunnels and associated pumps were bleached and cleaned weekly to reduce potential bacterial growth in the system.

Oxygen saturation of the water inside the swim tunnels was measured using mini fibre-optic oxygen probes (one per tunnel), which were continuously monitored and recorded by AutoResp software (version 2.2.2). The oxygen probes were connected to the AutoResp software via a Witrox-4 oxygen meter (Loligo, Denmark) for the two-tunnel system and via a Witrox-1 oxygen meter for the single tunnel. Oxygen probes were calibrated weekly using a two-point, temperature-paired calibration technique. Water velocity in the swim tunnels was generated using a DAQ-M data acquisition device and a VFD controller (models 4x and 12x; SEW Eurodrive). The velocity (precision of <1 cm s^−1^) for each tunnel was controlled remotely through the use of the AutoResp program. To minimize disturbance and experimenter influence on the fish, the swim tunnels were surrounded by black shade material, while infrared cameras (QSC1352W; Q-See, China), mounted directly overhead each tunnel, were connected to a television monitor and a DVR recorder to monitor individual fish behaviour.

Metabolic measurements for both routine and maximal oxygen uptake were made using intermittent respirometry (Cech, 1990; reviewed by Clark *et al*., 2013). A flush pump (model 2; Danner) for each swim tunnel circulated aerated water through the swim chamber, and was turned off automatically through AutoResp software and a DAQ automated respirometry system to seal the tunnel and measure the decline in oxygen concentration in the tunnel water attributable to fish respiration over a minimal period of 2 min. Oxygen levels were never allowed to fall below 80% of air saturation, and oxygen levels were restored within the swim tunnel after approximately 2–5 min once the flush pump resumed circulating water from the water bath.

Percentage saturation was converted to oxygen concentration ([O_2_], in milligrams of oxygen per litre) using the following formula:

$$\left[O_{2}\right]{=\;\%O}_{2}\text{Sat}/100\times \alpha \left(O_{2}\right)\times \text{BP},$$

where %O_2_Sat is the percentage oxygen saturation of the water read by the oxygen probes; α(O_2_) is the solubility coefficient of oxygen in water at the water temperature (in milligrams of oxygen per litre per millimetre of mercury); and BP is barometric pressure in mmHg.

Metabolic rate (MR in in milligrams of oxygen per kilogram per minute) for resting and swimming fish was calculated using the following formula:

$$\text{MR}=\left\{\left[{(O}_{2}(A)-O_{2}(B))\times V\right]\;\times \;M^{-1}\right\}\;\times \;T^{-1},$$

where O_2_(A) is the oxygen concentration in the tunnel at the beginning of the closed respirometry (in milligrams of oxygen per litre); O_2_(B) is the oxygen concentration in the tunnel at the end of the seal (in milligrams of oxygen per litre); *V* is the volume of water in the tunnel (in litres); *M* is the mass of the fish (in kilograms); and *T* is the duration of the closed respirometry (in minutes).

Values of *Q*_10_, the ratio of rates over a 10°C temperature range, were calculated for test temperatures using the following formula:

$$Q_{10}={\left({\mathrm{MR}}_{2}/{\mathrm{MR}}_{1}\right)}^{\left(10/T_{2}-T_{1}\right)},$$

where MR_2_ is the mean metabolic rate measured at *T*_2_; MR_1_ is the mean metabolic rate measured at *T*_1_; and *T*_2_ > *T*_1_.

#### Routine metabolic rate

Fish were first fasted for 24 h in individual 0.5 m × 1.0 m rectangular, flow-through holding tanks with aerated acclimation water from the same source as the acclimation tanks, before being transferred into a swim tunnel between 15.00 and 17.00 h. A 1 h acclimation period at their acclimation water temperature was followed by adjustment to the test temperature (between 12 and 26°C) at an incremental rate of 1°C each 30 min (2°C h^−1^). Automatic measurements of routine oxygen uptake began 30 min after the test temperature was reached and continued overnight using AutoResp software. Measurement periods were 2400 s in duration. Flush period durations were adjusted according to temperature to ensure adequate oxygen saturation in the chambers, with a longer flush for warmer temperatures. A small pump (mini DC 30A) mixed the water (<5 cm s^−1^) within the swim chamber to allow for continued oxygen exchange without eliciting fish movements. Measurements were discarded whenever the fish was visibly active on the video recording, which was rare. The mean of the lowest three values obtained at least 30 min after test temperatures were reached was used as the estimate of RMR. Given that the fish were visibly inactive and were given many hours to recover after minimal handling stress, we anticipated that RMR was not substantially higher than SMR. Indeed, Chabot *et al*. (2016) provided considerable data on a range of fish species to suggest that the RMR of inactive fish is likely to be <10% higher than SMR. As a result, AAS was estimated from MMR − RMR, while FAS was estimated from MMR/RMR.

#### Maximal metabolic rate

Measurements of RMR were completed between 08.00 and 09.00 h, and a modified critical swimming velocity (*U*_crit_) protocol was started to swim the fish until exhaustion and measure MMR. Water velocity in the swimming chamber was gradually increased from 0 to 30 cm s^−1^ over a period of ~2 min and remained at 30 cm s^−1^ for 20 min. After this period, water velocity was increased in increments of ~10% of the previous test velocity (i.e. 3 cm s^−1^ if the previous step ranged between 30 and 39 cm s^−1^, 4 cm s^−1^ if between 40 and 49 cm s^−1^, etc.) and held for 20 min or until the fish was exhausted and unable to swim. Active metabolism was measured towards the end of each velocity increment by sealing off the swim tunnel and recording the decrease in oxygen saturation without allowing the water air saturation to drop below 80%. When metabolic rate was high, multiple measurements were possible for a single velocity step. The highest metabolic rate measured during active swimming activity was taken as MMR. Between measurements, fresh water was flushed into the tunnel until oxygen saturation was >95%. When a fish stopped swimming and became impinged upon the back screen, water velocity was decreased to ~15 cm s^−1^ for 1 min, and the test velocity was gradually restored over a 2 min period. A fish was considered exhausted if it did not resume swimming after an impingement or if a second impingement occurred during the same velocity step. At this point, the time and velocity of failure were noted, the tunnel was flushed, and the water velocity was decreased to RMR conditions (<5 cm s^−1^) to allow the fish to recover.

A few fish were resistant to prolonged swimming and so a burst swimming protocol was used to elicit MMR. These fish were first given a 20 min rest period with water velocity <5 cm s^−1^ before being swum at 30 cm s^−1^ for 10 min. Afterwards, water velocity was rapidly (~10 s) increased above the prolonged swimming velocities (~60 cm s^−1^), which required the fish to burst swim to maintain station for a maximal duration of 30 s. The water velocity was decreased to 30 cm s^−1^ for 2 min, and this protocol was repeated for at least 5 min, and up to 10 min (i.e. two to six burst swims) until the fish became exhausted. Norin and Clark (2016) suggest that the peak MR of an exhausted fish is a reliable estimate of MMR. Again, exhausted fish were allowed to recover in RMR conditions for at least 1 h.

**Figure 1:**
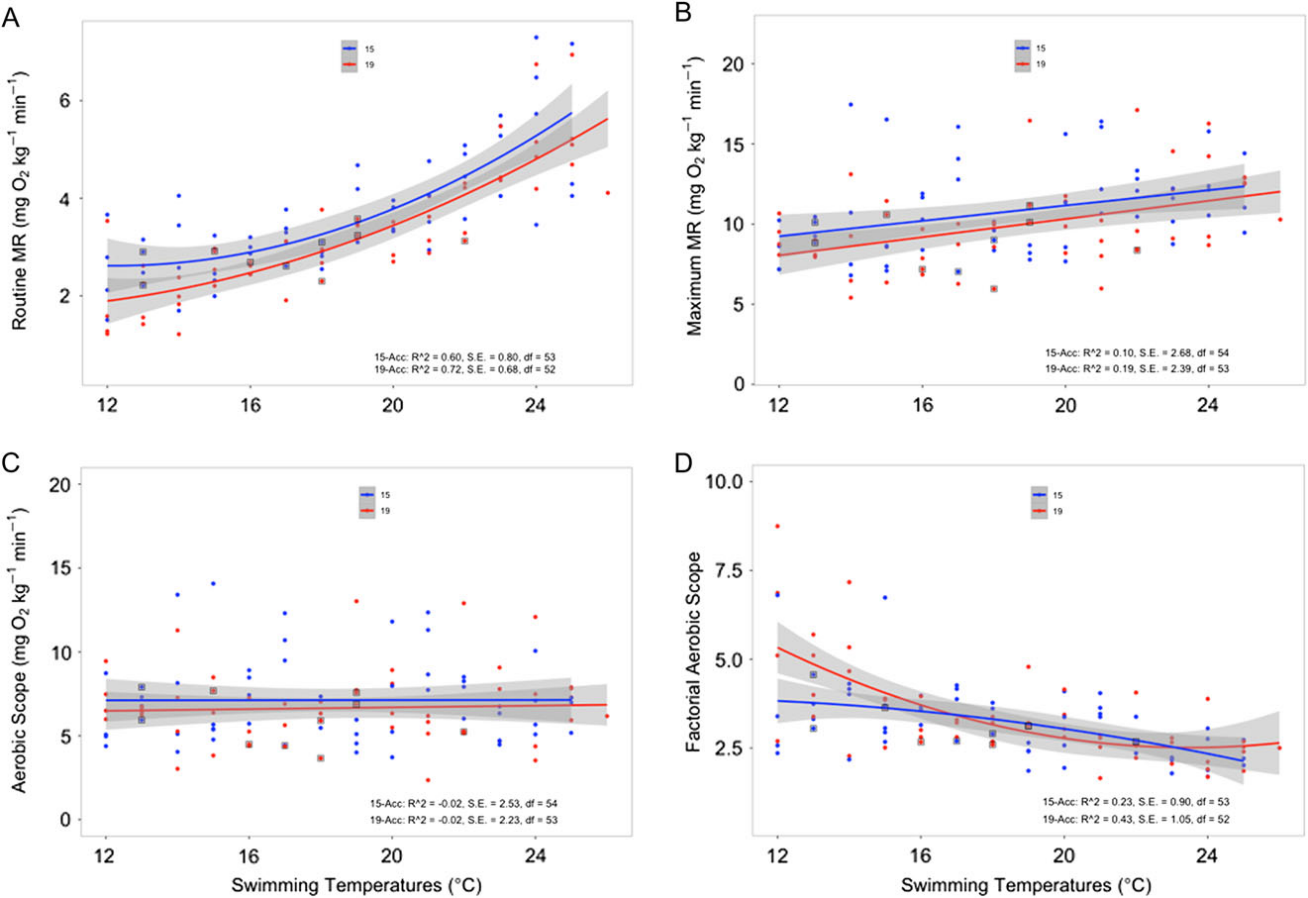
Thermal performance of hatchery juvenile Chinook salmon acclimated to 15 and 19°C, showing values of resting (routine) metabolic rates (RMRs; **A**), maximal metabolic rates (MMRs; **B**), absolute aerobic scope (AAS; **C**) and factorial aerobic scope (FAS; **D**). Each point represents one individual fish, and the continuous line represents the best-fitting line for the data; equations for best-fit curves are given in the text. The shaded area surrounding each line represents the standard error of the curve. ‘15-Acc’ and ‘19-Acc’ represent values for 15 and 19°C acclimation groups, respectively. Square boxes around individual data points represent individuals that were bursted to elicit MMR values (square boxes are indicated on RMR values to highlight which individuals required a bursting protocol). There was a significant effect of acclimation temperature on RMR values (*P* = 0.012), but no significant effect of acclimation temperature on MMR, AAS or FAS values (*P* > 0.05 for all comparisons). No data from fish that exhibited mortality were used to create the graphs.

Following the recovery period, water temperature was returned to the acclimation temperature at 2°C h^−1^ before fish were removed from the swim tunnel and placed for 24 h in a recovery tank. Following the 24 h recovery, the mass (in grams), fork length (in centimetres) and total length (in centimetres) were measured and recorded. Fish were then transferred to a designated long-term recovery tank. The absolute highest MMR value, regardless of the method of elicitation, was taken as the maximal rate. The few fish that required a burst swimming protocol to elicit MMR are included in the analysis and are highlighted in Fig. 1 to discriminate the data from those obtained using a modified *U*_crit_ protocol.

### Statistical analysis

Data were analysed using R Studio version 2.15.2 software (R Core Team, 2012) and the *car* (Fox and Weisberg, 2011), *plyr* (Wickham, 2011) and *multcomp* packages (Hothorn *et al*., 2008), while data were visualized using *ggplot2* (Wickham, 2009). Metabolic responses (RMR, MMR, AAS and FAS) were each analysed independently as a function of acclimation temperature and swimming temperature using a generalized linear model and subsequent *F*-value significance tests, and model fit was evaluated graphically. The effect of tunnel system on all metabolic rate values was found to be non-significant and not included in the final analysis. Relationships between test temperature and metabolic responses (RMR, MMR, AAS and FAS) were analysed for each acclimation group independently by modelling metabolic response as a function of test temperature. Two relationships between metabolic response and temperature were evaluated: linear and quadratic. The model with the highest adjusted *R*^2^ value and lowest residual squared error was chosen as being the best-fitting model. Significance was considered at α ≤ 0.05.

## Results

### Mortality

With the exception of one fish that died at 18°C, fish mortality during testing was limited to measurements at test temperatures of 25 and 26°C (Table 1). No post-test mortality occurred for fish that successfully survived RMR and MMR measurements. When fish were tested at 25°C, one out of four of the fish acclimated at 15°C died, but none of the fish acclimated at 19°C died at this temperature. When fish were tested at 26°C, mortality was higher; three out of four fish acclimated at 15°C (the other fish lost equilibrium but was revived) and three out of four fish acclimated at 19°C died during RMR measurements. None of the data from fish experiencing mortality were included in the subsequent analysis. The data obtained from the surviving fish at temperatures at which mortality occurred (25 and 26°C) are shown graphically for illustrative purposes only.

**Table 1:** Summary of all experiments performed

Acclimation temperature (°C)	Swimming temperature (°C)	Mortality (*n*)	Tested (*n*)	Bursted (*n*)
15	12	0	4	0
15	13	0	4	2
15	14	0	4	0
15	15	0	5	0
15	16	0	4	0
15	17	0	4	1
15	18	0	4	1
15	19	0	4	0
15	20	0	4	0
15	21	0	4	0
15	22	0	4	0
15	23	0	4	0
15	24	0	4	0
15	25	1	4	1
15	26	3 (1 LOE)	4	0
19	12	0	4	0
19	13	0	4	0
19	14	0	4	0
19	15	0	4	1
19	16	0	4	1
19	17	0	4	0
19	18	1	5	1
19	19	0	4	2
19	20	0	4	0
19	21	0	4	0
19	22	0	4	1
19	23	0	3	0
19	24	0	4	0
19	25	0	4	0
19	26	3	4	0

Mortality refers to the number of fish (*n*) that died at any point in the experiment, and no values from experiments that resulted in mortality or loss of equilibrium (LOE) were used to calculate metabolic rates. The number of fish that required a bursting protocol (Bursted) to obtain maximal metabolic rates is also shown.

### Metabolic measurements

Comparisons of data at 12 and 23°C and the corresponding *Q*_10_ values are made for means calculated from fish tested at each temperature and not derived from fitted regression lines.

#### Routine metabolic rate

Routine metabolic rate increased with test temperatures from 12 to 25°C for both acclimation groups of juvenile Chinook salmon (Fig. 1A). Routine metabolic rate was significantly affected by both acclimation temperature (d.f. = 1, *F* = 6.67, *P* = 0.012) and test temperature (d.f. = 14, *F* = 15.7, *P* < 0.001), but there was no significant interaction between the two (d.f. = 13, *F* = 0.47, *P* = 0.94). The RMR was lowered by warm acclimation, and this response was independent of test temperature, although more pronounced at lower test temperatures. However, mean RMR for fish acclimated and tested at 15°C (2.58 ± 0.22 mg O_2_ kg^−1^ min^−1^) was similar to that measured for fish acclimated and tested at 19°C (3.47 ± 0.07 mg O_2_ kg^−1^ min^−1^).

The response of RMR of fish acclimated at 15°C to test temperature (Fig. 1A) was fitted (*P* < 0.0001) with the following relationship: RMR (mg O_2_ kg^−1^ min^−1^) = 2.643 − 0.046*x* + 0.019*x*^2^. For fish acclimated at 15°C, mean RMR was 2.52 ± 0.47 mg O_2_ kg^−1^ min^−1^ when tested at 12°C and increased to 5.12 ± 0.37 mg O_2_ kg^−1^ min^−1^ when tested at 23°C, which is a *Q*_10_ of 1.91 over an 11°C temperature range. The response of RMR of fish acclimated at 19°C to test temperature (Fig. 1A) was fitted (*P* < 0.0001) with the following relationship: RMR (mg O_2_ kg^−1^ min^−1^) = 1.81 − 0.071*x* + 0.012*x*^2^. For fish acclimated at 19°C, mean RMR was 1.90 ± 0.55 mg O_2_ kg^-1^ min^−1^ when tested at 12°C and increased to 4.76 ± 0.36 mg O_2_ kg^−1^ min^−1^ when tested at 23°C, which is a *Q*_10_ of 2.30 over an 11°C temperature range.

#### Maximal metabolic rate

The MMR of juvenile Chinook salmon increased linearly as test temperatures increased from 12 to 25°C for both acclimation groups (Fig. 1B), although this increase did not reach statistical significance (d.f. = 14, *F* = 1.68, *P* = 0.08). Likewise, MMR was unaffected by acclimation temperature (d.f. = 1, *F* = 3.01, *P* = 0.09), and there was no significant interaction (d.f. = 13, *F* = 1.58, *P* = 0.11) between the two. Thus, the effect of test temperature on MMR was similar for both acclimation groups. The mean MMR for fish acclimated and tested at 15°C was 9.64 ± 1.75 mg O_2_ kg^−1^ min^−1^ vs. 12.23 ± 1.43 mg O_2_ kg^−1^ min^−1^ for fish acclimated and tested at 19°C.

The response of MMR of fish acclimated at 15°C to test temperature (Fig. 1B) was fitted (*P* = 0.01) with the following relationship: MMR (mg O_2_ kg^−1^ min^−1^) = 9.01 + 0.239*x*. For fish acclimated at 15°C, the mean MMR was 8.31 ± 0.73 mg O_2_ kg^−1^ min^−1^ when tested at 12°C and increased to 10.69 ± 0.78 mg O_2_ kg^−1^ min^−1^ when tested at 23°C, which is a *Q*_10_ of 1.26 over an 11°C temperature range. The response of MMR of fish acclimated at 19°C to test temperature (Fig. 1B) was fitted (*P* < 0.001) with the following relationship: MMR (mg O_2_ kg^−1^ min^−1^) = 7.77 + 0.284*x*. For fish acclimated at 19°C, the mean of MMR was 9.25 ± 0.56 mg O_2_ kg^−1^ min^−1^ when tested at 12°C and increased to 11.93 ± 1.97 mg O_2_ kg^−1^ min^−1^ when tested at 23°C, which is a *Q*_10_ of 1.26 over an 11°C temperature range. Thus, the rate of increase in MMR with test temperature and the absolute values for MMR were similar to those obtained for fish acclimated to 15°C.

#### Absolute aerobic scope

The AAS estimated for juvenile Chinook salmon acclimated to 15 and 19°C did not change significantly with an increase in either test temperature from 12 to 25°C (Fig. 1C; d.f. = 14, *F* = 0.27, *P* = 0.99) or acclimation temperature (d.f. = 1, *F* = 1.07, *P* = 0.30), and there was no interaction (d.f. = 13, *F* = 1.69, *P* = 0.08). Thus, AAS was independent of the test and acclimation temperatures used in this study. Mean AAS for fish acclimated and tested at 15°C was 7.06 ± 1.76 mg O_2_ kg^−1^ min^−1^ vs. 8.80 ± 1.42 mg O_2_ kg^−1^ min^−1^ for fish acclimated and tested at 19°C.

No clear peak in AAS was observed with test temperatures up to 23°C because the response of AAS of fish acclimated at 15°C to test temperature (Fig. 1C) was fitted with the following non-significant (*P* = 0.99) relationship: AAS (mg O_2_ kg^−1^ min^−1^) = 7.11 + 0.002*x*. For fish acclimated at 15°C, the mean AAS was 5.79 ± 0.99 mg O_2_ kg^−1^ min^−1^ when tested at 12°C and decreased to 5.57 ± 0.57 mg O_2_ kg^−1^ min^−1^ when tested at 23°C, which is a *Q*_10_ of 0.97 over an 11°C temperature range. As with the fish acclimated at 15°C, no clear peak in AAS for fish acclimated to 19°C was observed because the response of AAS to test temperature (Fig. 1C) was fitted with the following non-significant (*P* = 0.72) relationship: AAS (mg O_2_ kg^−1^ min^−1^) = 6.46 + 0.026*x*. For fish acclimated at 19°C, the mean AAS was 7.35 ± 0.76 mg O_2_ kg^−1^ min^−1^ when tested at 12°C and decreased to 7.18 ± 1.30 mg O_2_ kg^−1^ min^−1^ when tested at 23°C, which is a *Q*_10_ of 0.98. Thus, the absolute values for AAS and the independence of test temperature were similar to those obtained for fish acclimated to 15°C.

#### Factorial aerobic scope

The factorial aerobic scope of both acclimation groups decreased as test temperatures increased (Fig. 1D). Test temperature significantly affected FAS (d.f. = 14, *F* = 4.02, *P* < 0.0001), but FAS was not significantly affected by acclimation temperature (d.f. = 1, *F* = 1.32, *P* = 0.25), and there was no significant interaction (d.f. = 13, *F* = 1.44, *P* = 0.16). Although FAS decreased with test temperatures independent of acclimation temperature, the rate of decrease in FAS was similar for the two groups, except at temperatures lower than roughly 15°C (Fig. 1D). This is reflected in higher FAS values for fish acclimated at 19°C at cooler test temperatures. Mean FAS, however, for fish acclimated and tested at 15°C was the same (3.8 ± 0.75) as that measured for fish acclimated and tested at 19°C (3.6 ± 0.41).

The response of FAS of fish acclimated at 15°C to test temperature (Fig. 1D) was fitted (*P* < 0.001) with the following relationship: FAS = 3.87 − 0.034 − 0.006*x*^2^. For fish acclimated at 15°C, the mean FAS was 3.8 ± 1.0 when tested at 12°C and decreased to 2.1 ± 0.1 when tested at 23°C. The response of FAS of fish acclimated at 19°C to test temperature (Fig. 1D) was fitted (*P* < 0.0001) with the following relationship: FAS = 5.84 − 0.532 + 0.021*x*^2^. For fish acclimated at 19°C, FAS was 5.85 ± 1.3 when tested at 12°C and decreased to 2.5 ± 0.2 when tested at 23°C. The relatively high FAS values observed for fish acclimated at 19°C and tested below 15°C were driven by their very low RMR values. However, the differences in the loss of FAS between acclimation groups disappeared as temperatures increased, and suggests that the two acclimation groups were affected in a similar manner by changes in test temperature above 15°C.

## Discussion

The results from this experiment were intended to represent a range of responses to environmentally relevant acclimation temperatures (15 and 19°C). Likewise, the acute temperature changes (from 12 to 26°C) encompass the cooler temperatures experienced by juvenile Chinook salmon in upper tributary rearing grounds (10–14°C; Sacramento River Temperature Task Group, 2014) and warmer temperatures potentially experienced in the Sacramento–San Joaquin Delta (~25°C; Interagency Ecological Program Environmental Monitoring Program, 2015) as they migrate. While providing valuable new information about the thermal performance of Chinook salmon, caution is always needed when applying data from hatchery fish tested in the laboratory to their wild counterparts, although some evidence suggests that these physiological capabilities can be similar for fish tested in the field and the laboratory (Lee *et al*., 2003a). Nevertheless, the capability of juvenile Chinook salmon from both acclimation groups to perform with acute warming up to 23°C was unexpected.

Indeed, juvenile Chinook salmon performed in a similar manner in swimming tests and had similar estimated aerobic capacities at both acclimation temperatures (15 and 19°C), as well as over a range of test temperatures up to 23°C. Fish maintained AAS by matching the increase in RMR up to 23°C with an equivalent increase in MMR. Therefore, we conclude that AAS in this population of juvenile Chinook salmon shows a large degree of thermal independence at test temperatures extending above 20°C. Thus, experiments examining acclimation temperatures >19°C are certainly warranted for this stock of Chinook salmon. Thermal insensitivity of AAS has been documented previously in another Californian *Oncorhynchus* species; hatchery *Oncorhynchus mykiss* aerobic scope was maintained between 16 and 25°C (Verhille *et al*., 2016), and wild-caught *O. mykiss* tested on the Lower Tuolumne River was similar (Verhille *et al*., 2015). Again, *O. mykiss* mortality was evident post-exercise only at 25°C. Thus, the possibility that the *Oncorhynchus* genus located in the Central Valley of California may generally lack a clear intermediate peak of thermal performance needs to be explored, because it contrasts with intermediate thermal optima and peak performance in aerobic scope for more northerly sockeye salmon (*Oncorhynchus nerka*; Eliason *et al*., 2011; 2013). Although a peak or plateau in AAS is predicted by the OCLTT hypothesis (Pörtner and Knust, 2007; Pörtner and Farrell, 2008), this peak is so skewed to high temperatures that it is difficult to measure, which is what Fry showed for some of the many fish species that he studied (see Farrell, 2009).

Given the unexpected results of thermal insensitivity in aerobic scope, it is important to examine the quality of the data. The methods used in the present study provided an estimate of true AAS by using RMR instead of SMR (see Chabot *et al*., 2016). However, if the true AAS was underestimated, it was likely to be by no more than 10–15% because we visually eliminated fish activity during the overnight recovery and measurement periods. Also, we have no reason to suspect that this error varied in a systematic fashion across test temperatures. The MMR was almost always estimated based on the performance in an incremental swimming test, as prescribed by Fry (1971). Those tests that used burst swimming to exhaustion to estimate MMR were limited in number and yielded data that could not be distinguished from the results for incremental swimming (these data are identified in the figures). Therefore, our observations lend limited support to the suggestion of Norin and Clark (2016) that MMR can be measured using both approaches. A comparison of literature values for absolute values of AAS among juvenile salmonids is made in Table 2. Even though such comparisons are potentially confounded by the difference in fish body mass among the various studies, it is clear that the aerobic scope values obtained for juvenile Chinook salmon are similar and even slightly higher than some populations, especially juvenile *O*. *mykiss* (i.e. Verhile *et al*., 2015). Thus, qualitatively the AAS data do not seem suspect.

**Table 2:** Comparison of laboratory-derived absolute aerobic scope across juvenile *Oncorhynchus* spp.

	Population			Temperature (°C)			
Species	[Wild (W), Hatchery (H)]	Mass (g)	TL (cm)	Acclimation	Test	AAS (mg O_2_ kg^−1^ min^−1^)	Reference
*O. tshawytscha*	CVFR (H)	19.8 ± 0.02	12.0 ± 0.01	15	15	7.06 ± 1.76	Present study
*O. tshawytscha*	CVFR (H)	19.8 ± 0.02	12.0 ± 0.01	19	19	8.80 ± 1.42	Present study
*O. mykiss*	LTR (W)	22.4 ± 1.8	12.6 ± 0.3	Unk.	15	5.10	Verhille *et al*. (2015)
*O. mykiss*	Ontario (H)	6	n.a.	15	15	5.8	Scarabello *et al*. (1992)
*O. mykiss*	BC (W)	92.0 ± 11	15–20	12–24	13	5.83*	Gamperl *et al*. (2002)
*O. mykiss*	LBR (W)	58.0 ± 6.0	15–20	12–18	24	8.38*	Gamperl *et al*. (2002)
*O. mykiss*	BC (W)	108.0 ± 12	15–20	12–24	13	8.2*	Gamperl *et al*. (2002)
*O. mykiss*	LBR (W)	71.0 ± 5.0	15–20	12-12	24	7.3*	Gamperl *et al*. (2002)
*O. mykiss*	12M (W)	56.5 ± 3.8	n.a.	19–30	24	9.48*	Rodnick *et al*. (2004)
*O. mykiss*	RC (W)	50.4 ± 2.9	n.a.	12–27	24	9.23*	Rodnick *et al*. (2004)
*O. mykiss*	BC (W)	62.9 ± 5.6	n.a.	13–21	24	7.71*	Rodnick *et al*. (2004)
*O. nerka*	W	37	17	5	5	6.7	Brett (1964)
*O. nerka*	W	33	16	10	10	7.3	Brett (1964)
*O. nerka*	W	35	19	15	15	12.5	Brett (1964)
*O. nerka*	W	63	19	20	20	11	Brett (1964)
*O. nerka*	W	52	18	24	24	11.9	Brett (1964)

Abbreviations: AAS, absolute aerobic scope; BC, Bridge Creek; CVFR, Central Valley, CA autumn-run; LBR, Little Blitzen River; LTR, Lower Tuolumne River; 12M, 12 Mile; RC, Rock Creek; SR, Seymour River; TL, total length; Unk., unknown: fish were tested immediately after capture from the wild. *Values are expressed as milligrams of O_2_ per kilogram^−0.882^ per minute.

Despite this impressive aerobic capacity of juvenile Chinook salmon, some fish above 23°C were willing to exercise to a state from which they could not recover, a phenomenon first noted by Black (1957) when salmonids are exhaustively swum at an excessive temperature. Thus, like barramundi (Norin *et al*., 2014), pink salmon (*Oncorhynchus gorbuscha*; Clark *et al*., 2011) and common killifish (*Fundulus heteroclitus*; Healy and Schulte, 2012), juvenile Chinook salmon maintained maximal oxygen extraction from the water at temperatures that are very close to their limits of thermal tolerance. Why salmonids show post-exhaustion mortality following strenuous muscle activity at high temperature (Black, 1957; Parker and Black, 1959; Eliason *et al*., 2013) is unclear. However, this mortality has been associated with an increase in anaerobic effort (as measured by lactate accumulation in the plasma; Wilkie *et al*., 1997; Jain and Farrell, 2003), an increased ability or willingness to accumulate an oxygen debt (Lee *et al*., 2003b), or cardiorespiratory collapse attributable to limitations in scope for heart rate (Eliason *et al*., 2013). Literature on critical thermal maximum (CT_Max_) and upper incipient lethal temperature (Table 3) show that juvenile Chinook salmon experience mortality when exposed to or held at temperatures above 24°C. Given that CT_Max_ data for these fish range from 26.5 to 28.6°C (Muñoz *et al*., 2014; Fangue, N., Baird, S., and Cocherell, D., unpublished), exercise-induced mortality occurred below the CTmax, as might be expected. Moreover, the present population has the highest reported CT_Max_ (28.6°C), which could indicate a local thermal adaptation for this autumn-run population of Chinook in California. Indeed, they are currently the most abundant of the population segments located in the Central Valley of California (National Marine Fisheries Service, 2009), which is the southern-most portion of their native distribution. Fish in the *Oncorhynchus* genus have shown the ability to adapt thermally to local conditions, even when the temperatures encountered are well above those in their native range (i.e. *O. mykiss* in Western Australia; Molony *et al*., 2004; Chen *et al*., 2015), and local thermal adaptation in Chinook populations is possibly similar.

**Table 3:** Thermal tolerance of juvenile Chinook salmon

Population						
[Wild (W), Hatchery (H)]	Mass (g)	TL (cm)	Acclimation Temperature (°C)	Thermal measurement	Value (°C)	Reference
CVFR (H)	5.9 ± 0.06	8.6 ± 0.04	12	CT_Max_	28.6 ± 0.04	Fangue, N., Baird, S., and Cocherell, D., unpublished
BCBQR (W)	3.6 ± 1.1	n.a.	10	CT_Max_	26.5 ± 1.0	Muñoz *et al*. (2014)
WA (H)	1.03 ± 0.3	4.4 ± 0.4	20	UILT	25.1	Brett (1952)
WA (H)	1.03 ± 0.3	4.4 ± 0.4	24	UILT	25.1	Brett (1952)
BCBQR (H)	0.44 ± 0.05	3.9 ± 0.05	n.a.	LT_50_	24.7	Brett *et al*. (1982)
H	n.a.	n.a.	21.1	UILT	24.9	Orsi (1971)

Values represent means ± SEM. Abbreviations: BCBQR, British Columbia, Big Qualicum River; CT_Max_, critical thermal maximum; CVFR, Central Valley, CA autumn-run; LT50, median lethal temperature; n.a., not available; UILT, upper incipient lethal temperature; WA, Dungeness, Washington.

The lack of difference in performance between acclimation groups was perhaps unsurprising, and may indicate the ability to maintain homeostasis through physiological plasticity. Physiological plasticity in the form of thermal acclimation is well documented for Chinook salmon. In terms of acute thermal responses, Chinook salmon increased heat-shock protein 90 expression in heart, muscle, brain and gill tissues after a 5 h exposure to 21.6°C, indicating an acute compensatory mechanism (Palmisano *et al*., 2000). Furthermore, this thermal tolerance of juvenile Chinook salmon is reflected to some degree in their growth performance. Juvenile autumn-run Chinook reared at fluctuating temperatures of 13–16, 17–20 or 21–24°C still survived and grew at temperatures up to 24°C, albeit at significantly reduced rates at 24°C (Marine and Cech, 2004). The new finding that AAS and FAS for fish acclimated at 19°C tested at their acclimation temperature were similar to those values obtained for fish acclimated at 15°C tested at their acclimation temperature suggests that the optimal acclimation temperature range for this stock of juvenile Chinook salmon might lie between or even outside of these two temperatures. The life-history strategy of Chinook salmon exposes juveniles to both cooler riverine temperatures, associated with rearing grounds located in the upper reaches of the watershed, and warmer water temperatures in the bays and estuaries through which they must swim as they migrate to the ocean. Owing to the wide range of natural temperatures experienced by juvenile Chinook salmon, both 15 and 19°C could be important temperatures for this species to maintain physiological performance, leading to the observed lack of difference in performance between these acclimation groups. Although we note the tendency of AAS and MMR to be lower for fish acclimated at 19 than at 15°C across the test temperature range, further testing at acclimation temperatures <15°C and >19°C may be needed to define the optimal acclimation temperature for AAS.

Quantifying physiological performance in response to environmental variables is crucial for implementing effective conservation and management actions and for elucidating the mechanistic links between the environment and larger-scale processes, such as changes in population levels (reviewed by Horodysky *et al*., 2015). Furthermore, these investigations can help to guide future research efforts and inform management targets. For example, the inter-individual variation observed in MR measurements and AAS values in the present study and in previous work (Millidine *et al*., 2009; Norin and Malte, 2012; Metcalfe *et al*., 2016) warrants further investigation. This variation could be due to relaxed selective pressure associated with hatchery populations, which would allow for the survival of individuals with potentially low fitness (reviewed by Reisenbichler and Rubin, 1999) and low physiological performance capabilities. A comparison of thermal performance with wild populations would be necessary to determine whether the observed variation is attributable to hatchery practices. Conversely, the observed variation could be an adaptive trait of this population of Chinook salmon and could indicate adaptive potential. Variation in physiological performance among salmon populations allows for increased probability of population persistence in the face of environmental variability (i.e. the portfolio effect; Hilborn *et al*., 2003; Moore *et al*., 2010; Schindler *et al*., 2010). This same concept may apply to within-population variation as well. If environmental conditions shift or change rapidly, variation in individual physiological capacity indicates that some proportion of the population could survive and reproduce in the new environment. Thus, the variation observed in the present study may indicate that autumn-run Chinook salmon have the ability to persist as climate change occurs and that management actions may be effective even as temperatures continue to rise. Likewise, inter-individual variation within a population allows for specific management targets to be set; a specific subset of the population could be targeted for conservation and management if deemed appropriate. Therefore, quantifying not only physiological performance but also the variation in that performance is crucial for future effective management of salmonids.

Importantly, autumn-run Chinook salmon reside within the Sacramento–San Joaquin watershed, a heavily modified system with altered and managed flow regimes. For example, the altered temperature regime in the Sacramento River is the result of water diversions, impoundments, habitat modification and structured water releases focused towards the conservation of winter-run Chinook salmon, spring-run Chinook salmon, California Central Valley steelhead and the Southern Distinct Population Segment of green sturgeon (*Acipenser medirostris*; National Marine Fisheries Service, 2009; Sacramento River Temperature Task Group, 2014). It is possible, however, that the current artificially imposed thermograph may be differentially beneficial to species of conservation concern, and it has been hypothesized that these species may have conflicting water temperature requirements. In order to set regulatory criteria that will adequately improve thermal habitat for a larger suite of native fishes, a thorough understanding of thermal physiology and aerobic capacity is necessary for the specific populations of the fishes in question. Studies of aerobic capacity can be used in concert with studies of other temperature-mediated impacts on populations to develop and evaluate regulatory criteria designed to protect native fishes. Notably, recent work completed by Verhille *et al*. (2015, 2016) has challenged the use of a single thermal regulatory criterion for a species across its entire range of habitats and populations, and argued for local thermal adaptation in aerobic capacity for a population of *O. mykiss*. Given the immense effort that is exerted to manage the thermal hydrograph in many heavily altered systems, it is crucial that data on physiological capability are integrated more effectively into the regulatory criteria driving management actions, and we argue that more consideration should be given to potential local adaption and variation between populations.

In conclusion, it is important that aerobic scope data are coupled with critically important temperature-mediated functions, such as heart rate, growth or swimming performance data, to gain a full understanding of how temperature affects and limits the habitat of juvenile salmonids. Future studies should include assessments of aerobic scope from different populations of Chinook salmon to test for local adaptation in time and/or space and to determine whether the inter-individual variation observed in the present study is conserved in wild populations of fish or attributable to the hatchery origin of the fish tested here. Hopefully, our results can be used to manage the early life-history stages of Chinook salmon better and to further our knowledge of how changing environmental conditions will affect native fish populations.
